# Tin-based Antitumour Drugs

**DOI:** 10.1155/MBD.1994.213

**Published:** 1994

**Authors:** Marcel Gielen

**Affiliations:** 1 Free University of Brussels V.U.B. Faculty of Applied Sciences Department of General and Organic Chemistry Room 8G512, Pleinlaan 2 Brussel B-1050 Belgium


### TIN-BASED ANTITUMOUR DRUGS

Marcel Gielen

Free University of Brussels V.U.B., Faculty of Applied Sciences,
Department of General and Organic Chemistry, Room 8G512, Pleinlaan 2, B-1050 Brussel, Belgium

We have been active in the synthesis and characterization of tin-based antitumour compounds for several years and we would like to summarize here the results that have already been patented[1] and that may therefore be disclosed[2].

We synthesized some diorganotin 2,6-pyridinedicarboxylates.The dimethyltin compound has been found inactive *in vitro* , whereas the di-n-butyltin, di-t-butyltin and diphenyltin compounds were found more active than cisplatin[2].

In order to increase the water-solubility of these diorganotin derivatives, which might be a way to increase the antitumour activity following Atassi[4], we converted them[5] into their tetraethylammonium halide adducts.

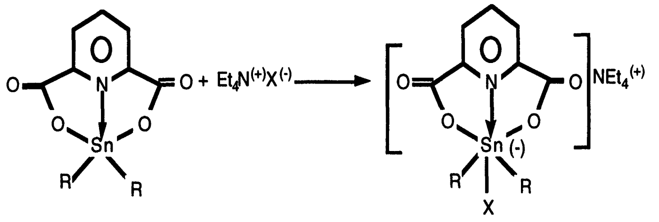

The *in vitro* antitumour activity of the tetraethylammonium halide adducts of these diorganotin 2,6-pyridine dicarboxylates (see table 11)[5] is not better than that of the parent molecules, even if their solubility in protic and also in less polar solvents is considerably enhanced.

We synthesized a series of di-n-butyltin derivatives of substituted salicylic acids and tested them against human tumour cell lines (see table 1)[2].

| | $ID_{50}$ values in ng/mL against | | | $ID_{50}$ values in ng/mL against | |
|---|---|---|---|---|---|
| Y | MCF-7 | WiDr | Y | MCF-7 | WiDr |
| 3-$CH_3$ | 44 | 330 | 4-$NH_2$ | 42 | 330 |
| 4-$CH_3$ | 51 | 316 | 5-$NH_2$ | 38 | 316 |
| 5-$CH_3$ | 90 | 337 | 5-COOH | 41 | 190 |
| 3-$CH_3O$ | 45 | 323 | 5-F | 46 | 256 |
| 4-$CH_3O$ | 190 | 1 794 | 5-Cl | 31 | 280 |
| 5-$CH_3O$ | 29 | 122 | 5-$SO_3H$ | 47 | 107 |
| Cisplatin | 850 | 624 | Mitomycin C | 3 | 17 |

*Table 1: $ID_{50}$ values (in ng/mL) of di-n-butyltin(IV) derivatives of substituted salicylic acids $[YC_6H_3(OH)COOSnBu_2]_2O\}_2$, and of cisplatin against MCF-7 and WiDr*

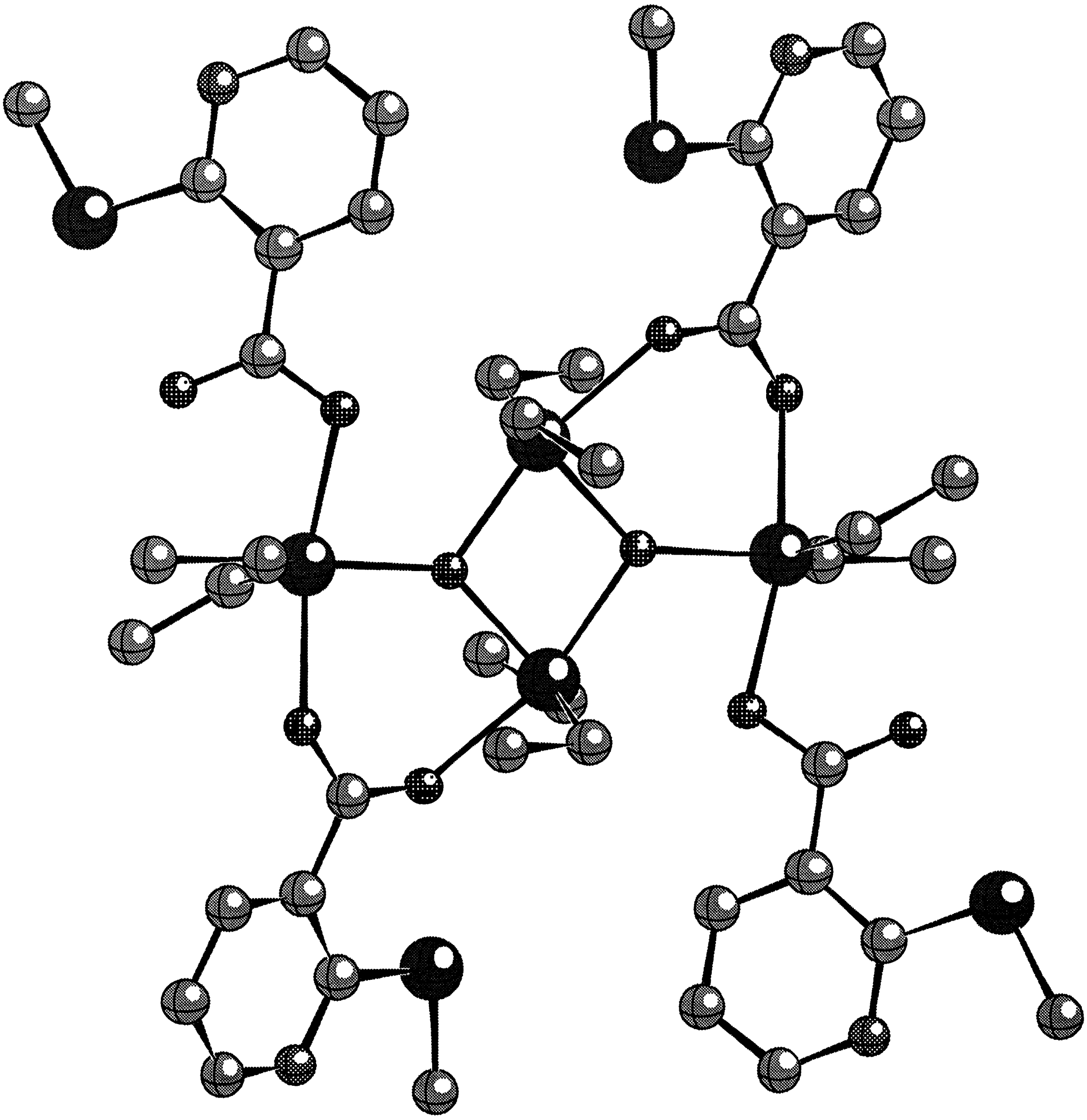

*Figure 1: X-ray structure of [diethyl(2-methylthio-3-pyridinecarboxylato) tin] oxide* [6]

Because Crowe has proposed that, among the factors relating the mode of action of diorganotin compounds $R_2SnX_2$, the organic groups R determine the potential activity[7], we prepared some diorganotin derivatives of substituted salicylic acids with various organic groups R linked to tin (see table 2)[2]. All the compounds of this type that we prepared were less active than the corresponding dibutyltin ones and than cisplatin.

| RR' | Y | $ID_{50}$ values in ng/ml against MCF-7 | WiDr |
|---|---|---|---|
| Me-n-Bu | 5-$CH_3O$ | 1 488 | 2 784 |
| $Et_2$ | 5-$CH_3O$ | 2 236 | 4 806 |
| n-$Oct_2$ | 5-$CH_3O$ | 4 677 | 10 639 |
| Cisplatin | | 850 | 624 |

*Table 2: $ID_{50}$ values of selected di-organotin(IV) derivatives of substituted salicylic acids, $\{[Y\text{-}C_6H_3(OH)COOSnRR']_2O\}_2$ and of cisplatin*

We also synthesized some other diorganotin 2,6-pyridinedicarboxylates, $C_5H_3N(COO)_2SnRR'$, varying once more the groups R and R' bound to tin (see table3). Here again, almost all the compounds prepared were less active than the di-n-butyltin derivative already described[2].

| | $ID_{50}$ values in ng/ml against | | | $ID_{50}$ values in ng/ml against | |
|---|---|---|---|---|---|
| RR' | MCF-7 | WiDr | | MCF-7 | WiDr |
| n-$Bu_2$ | 60 | 106 | Ph-i-Pr | 402 | 1 169 |
| [p-MeO-Ph]$_2$ | 4 930 | 15 800 | Ph-n-Bu | 761 | 3 705 |
| $Ph_2$ | 170 | 372 | Ph-i-Bu | 121 | 831 |
| PhMe | 2 187 | 3 283 | Ph[$PhCH_2$] | 2 910 | 10 995 |
| PhEt | 918 | 4 046 | Ph-[t-$BuCH_2CH_2$] | 50 | 161 |
| Ph-n-Pr | 223 | 1 094 | Ph[$PhMe_2CCH_2$] | 40 | 106 |
| Cisplatin | 850 | 624 | | | |

*Table 3: $ID_{50}$ values (in ng/ml) of selected 2,6-pyridinedicarboxylatodiorganotin(IV) derivatives $C_5H_3N(COO)_2SnRR'$ and of cisplatin*

Di-n-butyltin **di**carboxylates were also prepared, including some disalicylates[2] (see table 4). The 4-hydroxy-3-methoxybenzoate shows very high activities.

Several di-n-butyltin difluorobenzoates that we synthesized and characterized recently [9] exhibited very promising in vitro antitumour activities that are reported in table 5 together with $ID_{50}$ values on some compounds currently used clinically as antitumour agents are given for comparison.

From these data, it can be deduced that all the tested compounds score slightly better than cisplatin or etoposide against WiDr. Against MCF-7, they are even more active than doxorubicin, the 2,6-difluorobenzoate being as active as mitomycin C.

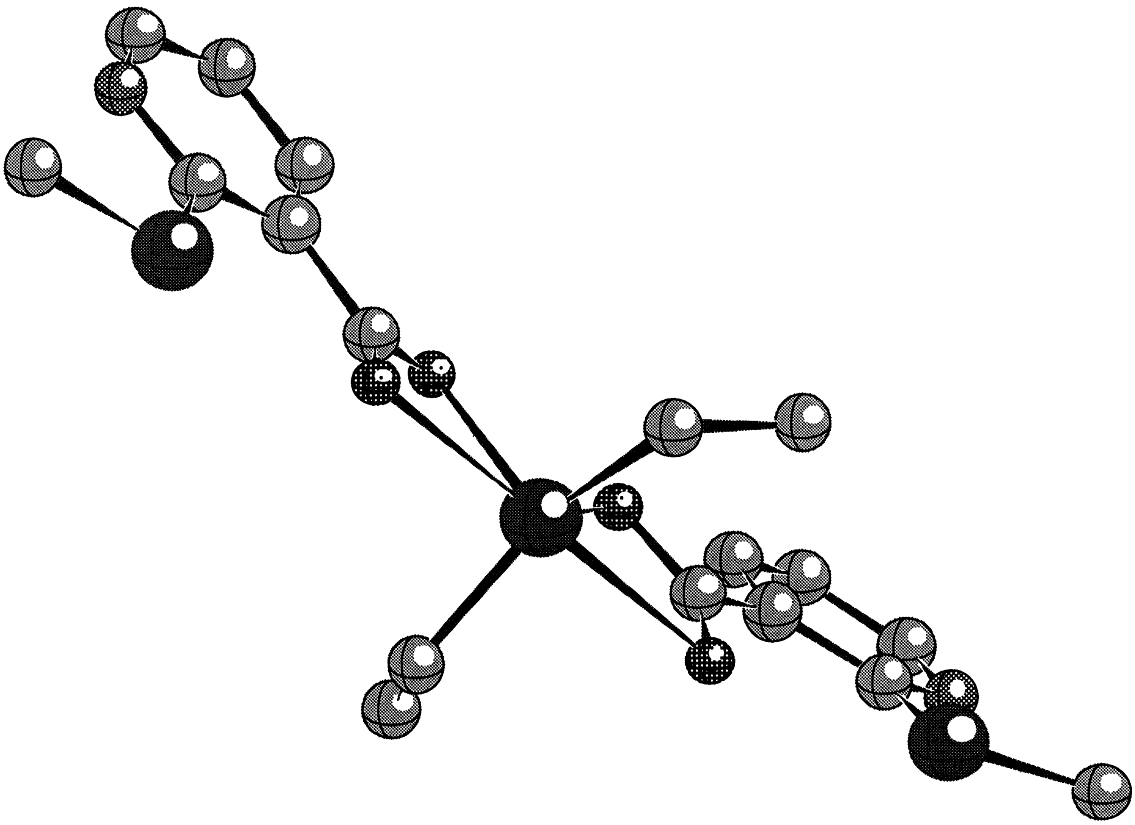

*Figure 2: X-ray crystal structure of diethyltin bis(2-methylthio-3-pyridinecarboxylate)* (6)

| X | Y | Z | MCF-7 | WiDr |
|---|---|---|---|---|
| H | H | 2-F | 74 | 242 |
| H | H | 3-F | 63 | 197 |
| H | H | 4-F | 90 | 309 |
| 2-OH | H | H | 541 | 2 974 |
| 2-OH | H | $3\text{-}CH_3O$ | 105 | 474 |
| 2-OH | H | $5\text{-}CH_3O$ | 54 | 611 |
| 2-OH | H | 5-Cl | 89 | 319 |
| 4-OH | H | $3\text{-}CH_3O$ | 44 | 82 |
| $3\text{-}OCH_3$ | $4\text{-}OCH_3$ | $5\text{-}OCH_3$ | 84 | 356 |
| $2\text{-}OCH_3$ | $3\text{-}OCH_3$ | $4\text{-}OCH_3$ | 93 | 398 |
| $2\text{-}OCH_3$ | $4\text{-}OCH_3$ | $5\text{-}OCH_3$ | 132 | 368 |
| Cisplatin | | | 850 | 624 |

*Table 4: $ID_{50}$ values (in ng/ml) of a series of diorganotin(IV) dicarboxylates $(X,Y,Z\text{-}C_6H_2COO)_2SnRR'$ and of cisplatin* (9)

| Molar ratio | Sunstituents | MCF-7 | WiDr |
|---|---|---|---|
| 1:2 | 2,3-$F_2$ | 23 | 283 |
| 1:2 | 3,5-$F_2$ | 30 | 407 |
| 1:1 | 2,3-$F_2$ | 9 | 120 |
| 1:1 | 2,5-$F_2$ | 7 | 277 |
| 1:1 | 2,6-$F_2$ | 3 | 174 |
| 1:1 | 3,5-$F_2$ | 11 | 172 |
| Cisplatin | | 850 | 624 |
| Etoposide | | 187 | 624 |
| Doxorubicin | | 63 | 31 |
| Mitomycin C | | 3 | 17 |

*Table 5: $ID_{50}$ values (ng/mL) of compounds of the type $(F_2C_6H_3COO)_2Sn(n\text{-}C_4H_9)_2$ (1:2 molar ratio), of the type $\{[(F_2C_6H_3COO)(n\text{-}C_4H_9)_2Sn]_2O\}_2$ (1:1 molar ratio), and of reference compounds tested against two human tumour cell lines, MCF-7 and WiDr*

We prepared also several original series of organotin molecules that are as active *in vitro* as mitomycin C against MCF-7 and WiDr. The first of these, that has recently been patented[1], are triphenyltin carboxylates[8].

| X | Y | Z | MCF-7 | WiDr |
|---|---|---|---|---|
| H | H | 2-$OCH_3$ | 16 | 15 |
| H | H | 4-F | 15 | 14 |
| H | 3-F | 5-F | 18 | 17 |
| H | 2-OH | 5-Cl | 11 | 18 |
| H | 2-OH | 5-$NH_2$ | 14 | 17 |
| H | 2-OH | 5-$OCH_3$ | 6 | 15 |
| 2-OH | 3-$CH(CH_3)_2$ | 5-$CH(CH_3)_2$ | 8 | 13 |
| Cisplatin | | | 850 | 624 |
| Mitomycin C | | | 3 | 17 |

*Table 5: Inhibition doses $ID_{50}$ in ng/mL against MCF-7 and WiDr obtained for a series of triphenyltin benzoates, $(C_6H_5)_3Sn\text{-}OOC\text{-}C_6H_2XYZ$ and for two reference compounds*

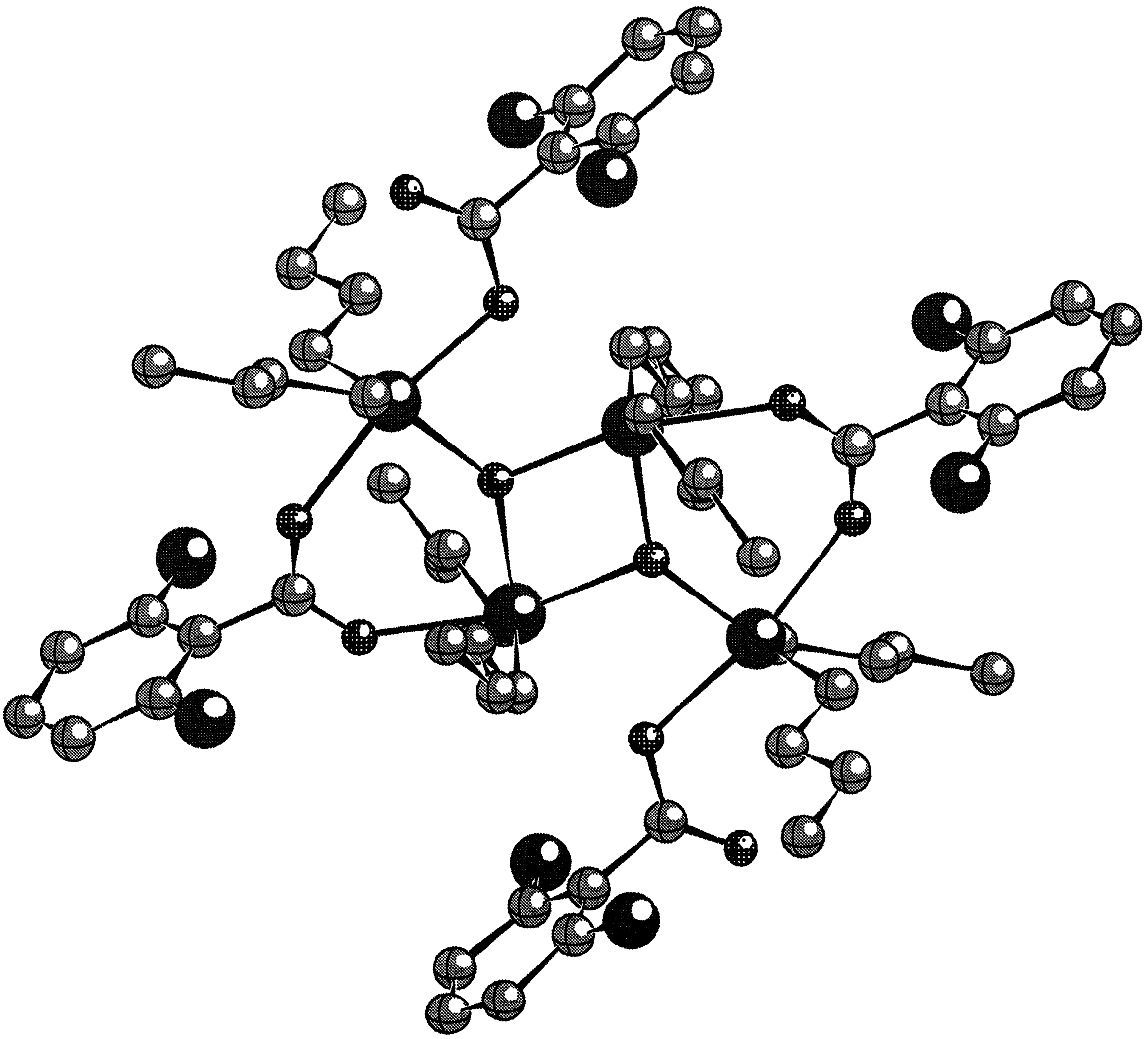

*Figure 2: X-ray crystal structure of [di-n-butyl(2,6-difluorobenzoato) tin] oxide* (9)

I hope that I have convinced you that several organotin compounds exhibit rather promising *in vitro* antitumour activities again human tumour cell lines. Of course we have to wait for the *in vivo* test results before claiming anything about the interest of such compounds for cancer chemotherapy. More work has to be done in the field of the preparation and testing of organotin molecules that might become useful antitumour drugs in the future.

### REFERENCES

(1) M. Bouâlam, M. Gielen, A. Meriem, D. de Vos and R. Willem (Pharmachemie B.V.): Antitumour compositions and compounds, *Eur. Pat.* 90202316.7-, 21/09/90; M. Bouâlam, M. Gielen, A. El Khloufi, D. de Vos and R. Willem, Pharmachemie B.V., Eur. Pat. 91202746.3-, 22.10.91, Novel organo-tin compounds having anti-tumour activity and anti-tumour compositions

(2) M. Gielen, P. Lelieveld, D. de Vos and R. Willem, *In vitro* antitumour activity of organotin compounds, in "Metal-Based Antitumour Drugs", vol. 2, Gielen M, ed., Freund Publ. House, Tel Aviv,1992, pp. 29 - 54 ; M. Gielen. P. Lelieveld, D. de Vos and R. Willem, "*In vitro* Antitumour Activity of Organotin(IV) Derivatives of Salicylic Acid and Related Compounds", in "Metal Complexes in Cancer Chemotherapy", Ed. B. Keppler, VCH, Weinheim, (1993), pp. 383 - 390

(4) G. Atassi, *Rev. Si, Ge, Sn & Sn Cpds*, **8** (1985), 219

(5) R. Willem, M. Biesemans, M. Bouâlam, A. Delmotte, A. El Khloufi and M. Gielen, *Appl. Organomet. Chem.*, 7 (1993), 311

(6) M. Gielen, A. El Khloufi, M. Biesemans, R. Willem and J. Meunier-Piret, Polyhedron, 11 (1992), 1861

(7) A.J. Crowe, The Antitumour Activity of Tin Compounds, in "Metal-Based Antitumour Drugs, vol 1, M. Gielen, Ed., Freund Publ. House Ltd, (1989), pp. 103 - 149

(8) M. Gielen, R. Willem, M. Biesemans, M. Bouâlam, A. El Khloufi and D. de Vos, *Appl. Organomet. Chem.*, **6** (1992), 287

(9) M. Gielen, M. Biesemans, A. El Khloufi, J. Meunier-Piret, F. Kayser and R. Willem, Diorganotin difluorobenzoates: synthesis, spectroscopic characterization and *in vitro* antitumour activity. X-Ray structure determination of bis[di-n-butyl(2,6-difluorobenzoato)tin] oxide, *J. Fluorine Chem.*, in press

**Received: September 2, 1993**

